# Editorial

**DOI:** 10.1017/qrd.2024.5

**Published:** 2024-02-06

**Authors:** Bengt Nordén

**Affiliations:** Department of Chemistry, Chalmers University of Technology, Gothenburg, Sweden

On the seventieth anniversary of the Watson and Crick paper ‘*Molecular Structure of Nucleic Acids: A Structure of Deoxyribose Nucleic Acid*’ in *Nature,* I would like to draw attention to a journal for original results: *QRB Discovery* that was launched by Cambridge University Press as a sister journal to *Quarterly Reviews of Biophysics* in 2020. Expertise is shared across the journals as they both have the same Editor-in-Chief and overlapping editorial boards.

Biophysics applies approaches and methods traditionally used in physics, chemistry, and mathematics to study the living world, from molecules and cells right up to populations of animals and plants. This interdisciplinary approach has a huge number of applications and has the potential to address some of the biggest challenges facing our species and our planet. It is vital that discoveries with the potential to benefit society are published quickly and transparently. The field has been missing a dedicated place to publish ground-breaking results – ‘discoveries’ – that point toward an exciting direction, rather than presenting a traditional comprehensive study. This is the gap *QRB Discovery* seeks to fill.

Why is the Watson and Crick paper relevant in this context? Well, *QRB* Discovery has an objective that well-established results (some might be from the literature) are used to underpin some important hypothesis – a speculation of great impact. Such a speculation is exactly what Watson and Crick put forward: based on available data on fibrous DNA, an intense X-ray diffraction at 3.4 Å consistent with stacked bases with planes orthogonal to the fiber axis, and one cross at 34 Å consistent with a helix of 10 base-pairs per turn and pitch 34 Å, they proposed a double helix in which adenine base was bound by two hydrogen bonds to a thymine, and guanine base to a cytosine base. That adenine would base-pair with thymine, and guanine with cytosine – both using two hydrogen bonds – was an educated guess based on stoichiometry with equal A and T contents (the exocyclic amino group of guanine forming a third hydrogen bond to cytosine – explaining specificity – was thus missed). Their speculated structure was proven correct, although first some 30 years later.

In *QRB Discovery*, authors are encouraged to elaborate on the potential consequences and wider impact of their discoveries. If the research is of high quality and it is a sound result that points in an exciting direction – even if that is speculative – we will publish. Some recent publications that really exemplify this are:

- ‘Origins of life: first came evolutionary dynamics’ (Charles Kocher and Ken A. Dill).
- ‘How sequence alterations enhance the stability and delay expansion of DNA triplet repeat domains’ (Jens Völker and Kenneth J. Breslauer).
- ‘On the micelle formation of DNAJB6b’ (Andreas Carlsson, Ulf Olsson, and Sara Linse).
- ‘Effect of relative humidity on hydrogen peroxide production in water droplets’ (Maria T. Dulay, Carlos Alberto Huerta-Aguilar, Christian F. Chamberlayne, Richard N. Zare, Adriaan Davidse, and Sinisa Vukovic). https://www.cambridge.org/core/journals/qrb-discovery/article/effect-of-relative-humidity-on-hydrogen-peroxide-production-in-water-droplets/ACC751343CBB32F8B1E2F5F954D36DFB

This transparency is further extended by publishing the open peer review reports. This will, expectantly, promote a more constructive type of review for authors but it will also contribute to the recognition of reviewers.

*QRB Discovery* has published a forward-looking collection entitled ‘Frontiers of Computational Biophysics’ (Guest Edited by Giulia Palermo), and has a number of other collections in the pipeline for 2024 – such as ‘Single Molecule Challenges in the 21st Century’ (Guest Edited by Felix Ritort, Fredrik Westerlund and Giulia Palermo) and ‘Perspectives in Integrated Biophysics: how to probe biological process with complementary multiscale techniques’ (Guest Edited by Alison Rodgers, Wah Chiu, and Sheemei Lok).

*QRB Discovery* is indexed in both PMID and PMCID immediately upon online publication. For example: https://pubmed.ncbi.nlm.nih.gov/34192260/.

I look forward to seeing what exciting research will be submitted next!

Wishing you all a prosperous 2024!

Bengt Nordén

## Editor’s Comment added in proof

For further inspiration read Norden B (2021). *Which are the ‘Hilbert Problems’ of Biophysics*? QRB Discovery, 2: e1, 1–3. https://doi.org/10.1017/qrd.2020.15.

